# Supplementary material for: A Mix of Dietary Fibres Changes Interorgan Nutrients Exchanges and Muscle-Adipose Energy Handling in Overfed Mini-Pigs
Source: Nutrients. 2021 Nov 23;13(12):4202. doi: 10.3390/nu13124202 (PMC8704711; doi:10.3390/nu13124202)
Supplement: Supplementary file 1 [file nutrients-13-04202-s001.zip › nutrients-1456492-supplementary.pdf]

Supplementary Table S1. Net flux (mmol/h) of ammonia, beta hydroxybutyrate (b-OH), the sum of the three short chain fatty acids (Σ SCFA), and amino acids across the portal drained viscera (PDV), liver and total splanchnic tissues (TSP) in the fasted state after 14 (D14) and 56 (D56) days of nutritional treatment.

		O			O+F			P value		
		D14	D56	SEM	D14	D56	SEM	Diet	Time	Diet x Time
Ammonia	PDV	6818	7716	733	8470	6638	867	0.81	0.54	0.10
	Liver	-6749	-7462	768	-9116	-6441	940	0.59	0.27	0.08
	TSP	419	868	454	-1999	-1272	497	0.13	0.18	0.74
b-OH	PDV	161	235	26	216	132	30	0.61	0.92	0.13
	Liver	-24	-10	57	28	69	62	0.52	0.85	0.93
	TSP	146	236	56	157	222	52	0.99	0.54	0.92
Σ SCFA	PDV	22674	20960	2291	34682	18318	2510	0.24	0.13	0.21
	Liver	-19115	-17255	2303	-20408	-10505	2303	0.46	0.23	0.40
	TSP	6737	7217	2253	13855	8554	2253	0.24	0.27	0.19
Leucine	PDV	92	403	184	-18	6	202	0.40	0.58	0.63
	Liver	-167	208	187	316	26	187	0.61	0.91	0.41
	TSP	-143	320	163	380	188	163	0.45	0.61	0.24
Isoleucine	PDV	0	218	112	-93	-36	122	0.35	0.52	0.70
	Liver	-39	137	134	159	24	134	0.84	0.94	0.59
	TSP	-107	138	89	142	122	89	0.41	0.50	0.44
Valine	PDV	497	41	395	-281	305	433	0.68	0.91	0.40
	Liver	-1043	883	497	667	-84	497	0.63	0.50	0.16
	TSP	-727	376	296	101	510	296	0.31	0.17	0.51
Lysine	PDV	321	360	135	-6	190	147	0.26	0.59	0.71
	Liver	-464	-505	196	-159	-592	196	0.72	0.35	0.43
	TSP	-57	-167	143	-13	-259	143	0.91	0.29	0.68
Phenylalanine	PDV	79	253	107	117	102	117	0.74	0.64	0.58
	Liver	-488	-296	134	-213	-361	134	0.61	0.89	0.31
	TSP	-232	-33	134	-53	-196	134	0.97	0.84	0.23
Methionine	PDV	37	72	38	18	-30	42	0.34	0.94	0.57
	Liver	-92	-262	88	-91	39	88	0.28	0.87	0.22
	TSP	-95	-229	61	-54	42	61	0.12	0.88	0.36
Threonine	PDV	-233	44	185	-356	23	203	0.81	0.41	0.90
	Liver	-207	-320	255	-64	-415	255	0.95	0.29	0.57
	TSP	-433	-342	188	-296	-271	188	0.72	0.79	0.88
Tryptophane	PDV	-27	-25	69	-60	-69	76	0.73	0.98	0.96
	Liver	-1	-56	90	5	-59	90	0.99	0.63	0.97
	TSP	214	147	181	-116	-41	181	0.36	0.98	0.69
Histidine	PDV	114	254	114	7	155	125	0.57	0.29	0.98
	Liver	-158	-333	116	-60	-176	116	0.48	0.36	0.85
	TSP	-193	-302	88	-40	72	88	0.07	0.99	0.43
Σ BCAA	PDV	590	662	662	-393	275	725	0.52	0.71	0.77
	Liver	-1249	1228	793	1142	-34	793	0.65	0.63	0.20
	TSP	-977	834	506	622	820	506	0.32	0.28	0.37
Σ indispensable AA	PDV	-3798	-2940	1201	-4941	-4674	1315	0.46	0.78	0.88
	Liver	-4219	-2099	1518	-639	-3604	1518	0.66	0.82	0.20
	TSP	-8103	-6221	1110	-5585	-7016	1110	0.61	0.86	0.22

Alanine	PDV	1489	1504	395	606	1538	433	0.50	0.21	0.22
	Liver	-3386	-3571	676	-1692	-2817	676	0.25	0.43	0.56
	TSP	-1878	-2149	429	-895	-1076	429	0.14	0.72	0.94
Glutamate	PDV	-1067	-267	249	89	-747	273	0.41	0.97	0.10
	Liver	5364	11405	1972	7749	10764	1972	0.77	0.09	0.53
	TSP	5625	10698	1852	8266	10107	1852	0.72	0.09	0.39
Glutamine	PDV	-2252	-2778	471	-3859	-1954	516	0.60	0.32	0.10
	Liver	2373	-2023	812	-20	-1162	812	0.55	0.07	0.25
	TSP	-1004	-4967	847	-3607	-2767	847	0.88	0.26	0.10
Glycine	PDV	872	4905	1630	-1323	4082	1786	0.56	0.01	0.63
	Liver	166	-4478	2149	25	-4712	2149	0.96	0.27	0.99
	TSP	-1500	-2002	1835	-958	169	1835	0.64	0.93	0.81
Proline	PDV	-526	604	434	-814	781	475	0.94	0.08	0.73
	Liver	-358	-351	350	770	-770	350	0.52	0.07	0.06
	TSP	-1376 *a*	-366	275	229 *b*	-125	275	0.06	0.60	0.29
Tyrosine	PDV	163	22	83	-50	29	91	0.44	0.80	0.38
	Liver	-451	-326	136	-207	-453	136	0.78	0.59	0.13
	TSP	-191	-227	105	-210	-350	105	0.65	0.34	0.57
Serine	PDV	-93	281	292	-411	-130	320	0.44	0.45	0.91
	Liver	-196	-501	192	-341	-406	192	0.93	0.63	0.76
	TSP	-459	-480	220	-448	-432	220	0.93	1.00	0.97
Arginine	PDV	207	59	89	-43	-66	97	0.21	0.64	0.73
	Liver	15	-354	309	-653	695	309	0.69	0.24	0.06
	TSP	136	-355	235	-409	696	235	0.49	0.43	0.06
Citrulline	PDV	1424	862	179	1036	1236	200	0.99	0.64	0.35
	Liver	-180	-462	296	202	-194	296	0.60	0.26	0.31
	TSP	1234	691	266	912	725	298	0.87	0.31	0.61
Cystine	PDV	66	68	73	-79	74	80	0.55	0.40	0.41
	Liver	-320	-29	113	-93	-75	113	0.82	0.86	0.71
	TSP	-283	-68	110	73	24	110	0.21	0.67	0.50
Ornithine	PDV	313	249	96	19	394	105	0.63	0.29	0.15
	Liver	-97	-66	119	-82	-164	119	0.82	0.86	0.71
	TSP	155	165	60	66	289	60	0.85	0.27	0.31
Asparagine	PDV	12	527	130	198	67	142	0.52	0.48	0.25
	Liver	-485	-979	126	-806	-604	126	0.90	0.66	0.31
	TSP	-73	-110	245	-571	-507	245	0.24	0.96	0.84
Aspartate	PDV	112	81	58	170	181	63	0.39	0.87	0.73
	Liver	-169	48	80	-95	-20	80	0.98	0.21	0.51
	TSP	-2	142	38	83	176	38	0.32	0.04	0.60
Taurine	PDV	654	-231	219	-176	106	239	0.50	0.53	0.25
	Liver	-616	-299	405	1	-208	405	0.57	0.90	0.56
	TSP	-41	-328	188	133	-19	188	0.41	0.38	0.78
Σ non indispensable AA	PDV	343	6267	2873	-4741	5204	3147	0.50	0.03	0.50
	Liver	2365	-1707	4338	4018	1821	4338	0.70	0.61	0.88
	TSP	153	1016	3780	1918	9020	3780	0.41	0.49	0.58
Σ AA	PDV	1222	7887	3972	-5413	5851	4351	0.49	0.07	0.60
	Liver	-295	-2252	5389	4578	224	5389	0.66	0.67	0.87
	TSP	-1619	925	4099	1969	9188	4099	0.36	0.44	0.71

Values are means P-values were obtained by Two Way Repeated Measures ANOVA; For a metabolite: a,b
At a same timepoint, O+F ≠ O.
